# Assessment of executive functions through a virtual reality task in euthymic patients with bipolar disorder and influence in psychosocial functioning

**DOI:** 10.1192/j.eurpsy.2023.1465

**Published:** 2023-07-19

**Authors:** J. Andreu Martínez, D. Beltrán Cristancho, P. Navalón, P. Sierra San Miguel, A. García Blanco, A. Chicchi Glioli, S. Cervera Torres, M. Alcañiz Raya, Y. Cañada Pérez

**Affiliations:** 1 Psychiatry, Hospital Universitario y Politécnico La Fe de Valencia; 2 Instituto de Investigación Sanitaria La Fe (Valencia), Valencia, Spain; 3 Psychiatry, Instituto de Investigación Sanitaria La Fe (Valencia); 4 Universidad Politécnica de Valencia (Valencia), Valencia, Spain

## Abstract

**Introduction:**

Previous research has shown that neurocognitive deficits, especially deficits in executive functions, may persist during euthymia in in patients with bipolar disorder (BD) and that those are associated with an impairment of psychosocial functioning. The assessment of executive functions (EFs) is normally carried out using laboratory tests. Novel methodologies such as virtual reality (VR) allow the creation of immersive environments, to evaluate executive performance with greater potential for ecological validity than evaluations with standard tasks.

**Objectives:**

The objectives of this project are to evaluate executive performance in euthymic patients with BD with a novel virtual reality task compared to standard computerized tasks, and to find predictors of functioning associated with cognitive performance.

**Methods:**

This is a cross sectional study in which 46 euthymic patients with BD treated at La Fe University and Polytechnic Hospital were assessed with a battery of standard computerized tasks (ST) (TMT/Stroop,/Go-No-Go/TOL/DOT) and with the Cooking Task virtual reality task. The Cooking Task presents 4 tasks of increasing difficulty in which you must cook food in a specific time. It records total time to complete the task, whether food is cooled or burned, the simultaneous use of two fires, the proper use of seasonings and the time to set the table.

Functioning was assessed with the Functioning Assessment Short Test (FAST) that evaluates the overall functioning of patients with a mental illness in 6 subscales.

Correlation analyses between cognitive performance variables and clinical variables were done. Multiple linear regression was performed with the FAST score as a dependent variable and cognitive performance variables and relevant clinical variables to executive functioning (months of euthymia, age, and number of total episodes) were included as independent variables.

**Results:**

A worse psychosocial functioning was significantly associated with a worse performance in standard tasks (TMTA, TMTB, STROOP, and TOL) and cooking task (total time spent on task 2, burning time and total time spent on task 3, and total time spent on task 4). In the regression analysis, the correct simultaneous use of the two fires was the best predictor of a better psychosocial functioning in BD patient. This implies the preserved ability of planning and performing dual tasks.

**Conclusions:**

Our findings suggest that euthymic patients with BD present deficits in executive functions related with a worse psychosocial functioning. Among the tasks, the cooking task may have a greater sensitivity than standards task to predict real functioning. This in an opportunity to design virtual applications for diagnostic and therapeutic purposes.

**Disclosure of Interest:**

None Declared

